# Reframing nature connectedness as a psychosocial mechanism for youth mental health

**DOI:** 10.3389/fpsyt.2026.1781920

**Published:** 2026-03-16

**Authors:** Fuling Liu, Zilai Zhang, Yanan Huang, Pengyu Wang, Zhihua Bao, Yiyue Nou, Zhen Zhang, Hongliang Sun, Tao Song

**Affiliations:** 1 Department of Mental Health and Psychology, Dalian Medical University, Dalian, China; 2 The Second Affiliated Hospital, Dalian Medical University, Dalian, China; 3 The First Affiliated Hospital, Dalian Medical University, Dalian, China; 4 College of Medical Imaging, Dalian Medical University, Dalian, China; 5 School of Stomatology, Dalian Medical University, Dalian, China; 6 College of Humanities, Dalian Medical University, Dalian, China; 7 College of Basic Medical Sciences, Dalian Medical University, Dalian, China

**Keywords:** adolescent mental health, nature connectedness, nature-based interventions, public health, social connectedness

## Abstract

Nature connectedness has emerged as an increasingly important resource for addressing the growing global challenge of youth mental health. Moving beyond exposure-based accounts of greenspace contact, this conceptual analysis reframes nature connectedness as a modifiable psychosocial mechanism in adolescent development. Integrating evidence and theory from developmental and environmental psychology, public health, and social science, we propose a three-pathway model through which nature connectedness may support mental health in young people: (i) emotional restoration (attentional recovery, stress buffering, and emotion regulation), (ii) behavioral regulation (physical activity, digital balance, and sleep), and (iii) social connectedness (belonging, community engagement, and family cohesion). This model provides a theoretically grounded and translational framework for designing, evaluating, and structurally embedding scalable, equity-oriented nature-based strategies within youth mental health systems.

## 1 Introduction

Adolescent mental health has emerged as one of the most urgent public health challenges worldwide (1, 2). Traditional mental health services, which rely heavily on clinical treatment and specialist care, remain insufficient to meet the scale of this growing burden (3). Structural barriers including limited access, high cost, social stigma, and workforce shortages further constrain the reach of formal mental health systems, leaving many adolescents without timely or appropriate support (4–6). This highlights the urgent need for preventive strategies that can strengthen mental health and resilience among adolescents.

Nature connectedness refers to the subjective sense of one’s relationship with the natural world, encompassing emotional attachment, cognitive identification, and relational belonging (7, 8). Its theoretical foundations are inherently interdisciplinary, drawing from environmental psychology, developmental psychology, public health, sociology, and ecological sciences (9). These perspectives conceptualize nature connectedness not merely as an environmental condition, but as a complex psychosocial construct that shapes perception, behavior, and social engagement across adolescence.

Within the field of mental health, growing evidence suggests that stronger nature connectedness is associated with lower levels of stress and emotional distress, improved mood, enhanced self-regulation, and greater overall well-being (10, 11). Importantly, nature connectedness is not a fixed personality trait but a malleable psychosocial capacity shaped by repeated experiences, environmental contexts, educational practices, and social norms (12). This plasticity is particularly relevant during adolescence, a period marked by heightened environmental sensitivity and ongoing reorganization of emotional and identity-related processes. In this sense, nature connectedness may represent a developmentally salient psychosocial resource.

However, despite the rapidly growing interest in nature connectedness, existing research remains fragmented and largely descriptive. Most studies document associations between nature connectedness and mental health outcomes without offering a coherent theoretical explanation of how and why this relationship operates, particularly within adolescent populations (13, 14). As a result, nature connectedness has not yet been systematically integrated into mainstream public health strategies for youth mental health promotion. While studies within environmental psychology have provided valuable insights into adolescents’ lived experiences, meanings, and emotional relationships with nature (15, 16), these findings have not yet been consolidated into a coherent, mechanism-oriented framework suitable for public health translation. Consequently, the potential of nature connectedness as a population-level mental health resource remains underutilized in policy design, program development, and preventive intervention models.

The present article aims to clarify this conceptual gap by examining how nature connectedness may be linked to adolescent mental health processes. While nature connectedness is commonly described in terms of emotional, cognitive, and relational dimensions, our focus is on outlining how these dimensions may operate through identifiable developmental pathways. Rather than redefining the construct, we seek to organize existing theoretical and empirical insights into a coherent framework. To this end, we present a three-pathway model that integrates emotional, behavioral, and social mechanisms through which nature connectedness may support youth wellbeing. This framework is intended to enhance conceptual clarity and to inform preventive public health applications.

## 2 Nature connectedness as a psychosocial determinant of youth mental health

The mental health benefits of exposure to natural environments are now widely recognized. However, prevailing public health frameworks often conceptualize nature primarily as an external environmental condition, operationalized through indicators such as green space availability, proximity to natural areas, or frequency of outdoor contact (17, 18). Although exposure-based approaches have generated important epidemiological insights, and qualitative research within environmental psychology has begun to illuminate how adolescents experience, interpret, and attribute meaning to nature, these lines of inquiry remain largely fragmented. Exposure metrics primarily capture measurable environmental contact, while qualitative studies offer rich contextual and experiential accounts. However, neither approach alone has yet provided a unified framework that systematically organizes the psychological, relational, and developmental processes through which human–nature interactions influence adolescent mental health. This fragmentation limits the translation of existing evidence into coherent public health strategies and scalable preventive interventions.

To address these limitations, we advance nature connectedness as a complementary and developmentally grounded conceptual perspective. Nature connectedness refers to an individual’s emotional affiliation, cognitive identification, and relational sense of belonging with the natural world (19, 20). Rather than replacing exposure-based approaches, this perspective focuses on how environmental contact becomes internalized as a relational psychological orientation that shapes perception, motivation, and social engagement.

Within this framework, nature connectedness is conceptualized not merely as an environmental correlate of mental health, but as a psychosocial determinant. Psychosocial determinants encompass the psychological, relational, and social factors that structure individuals’ mental health trajectories across the life course (21). These include emotional regulation, perceived support, coping resources, identity development, social connectedness, and the quality of interpersonal relationships (22). Unlike structural or purely biomedical determinants, psychosocial determinants operate primarily through subjective experience, interpretive processes, and patterns of interaction within social and environmental contexts (23). Nature connectedness aligns closely with this domain, exerting influence on mental health not simply through environmental presence, but through sustained modulation of emotional experience, behavioral regulation, and social integration. Importantly, this conceptualization does not exclude biological processes; rather, biological mechanisms such as stress physiology, autonomic regulation, and circadian functioning may be understood as dynamically interacting components within these psychosocial pathways.

This positioning is conceptually consistent with established public health systems frameworks, including the Social Determinants of Health model, the prioritization of social connection within population health, and the Social Exposome paradigm (24–26). These frameworks provide structural explanations for heterogeneity in health outcomes across populations. The present framework operates at a complementary level by specifying the relational and developmental mechanisms through which engagement with nature may organize emotional, behavioral, and social processes during adolescence.

Conceptually, nature connectedness is distinct from nature exposure, general wellbeing, and stable personality traits. It captures a relational orientation toward the natural world rather than a generalized positive disposition or an outcome-level state. Emerging relational accounts suggest that nature connectedness operates in ways analogous to interpersonal relationships, involving perceived mutuality, emotional significance, and incorporation of nature into the self-concept (19). In this sense, it reflects not merely contact with the environment, but an internalized pattern of engagement in which individuals experience nature as meaningful, identity-relevant, and psychologically significant. This distinction is important because wellbeing refers to multidimensional indicators of psychological functioning, whereas nature connectedness represents an upstream relational capacity that may shape those outcomes (27, 28). By conceptualizing nature connectedness as a relational and identity-relevant orientation, it becomes possible to explain how similar levels of environmental exposure may yield divergent psychological effects across adolescents. We therefore hypothesize that nature connectedness may offer complementary explanatory insights alongside exposure metrics and correlated psychosocial resources (e.g., baseline affect, trait mindfulness, physical activity propensity, and perceived social support).

Adopting this psychosocial perspective also helps clarify the marked heterogeneity observed in adolescents’ mental health responses to nature. Available evidence suggests that adolescents who report stronger emotional and relational bonds with nature exhibit greater resilience, lower perceived stress, and higher life satisfaction, whereas peers with comparable levels of environmental exposure but weaker subjective connectedness often show attenuated benefits (7, 29). These findings imply that it is not merely access to nature, but the quality of psychological engagement and relational experience that conditions mental health outcomes.

From a developmental psychology perspective, adolescence constitutes a sensitive period during which psychosocial influences exert particularly strong and enduring effects on mental health trajectories (30). This stage is marked by rapid transformations in emotional regulation, identity formation, cognitive maturation, and social reorientation (31, 32). During this window of heightened vulnerability and opportunity, adolescents actively seek stable sources of meaning, belonging, and self-definition. In this context, nature connectedness may function as a developmentally salient resource, providing a psychologically safe and low-pressure environment that supports emotional processing, identity exploration, and consolidation of self-concept.

From a public health perspective, conceptualizing nature connectedness as a psychosocial determinant also carries implications for health equity. Adolescents from socioeconomically disadvantaged backgrounds are disproportionately exposed to chronic stressors, including academic pressure, family instability, community violence, and limited access to formal mental health services (33). For these populations, nature connectedness represents a potentially accessible and culturally adaptable psychosocial resource, offering psychological relief and emotional support with relatively low structural and financial barriers (34). By strengthening emotional regulation, fostering adaptive coping, and enhancing social integration, nature connectedness holds promise for mitigating mental health disparities and promoting more equitable outcomes at the population level.

Taken together, this synthesis supports the proposition that nature connectedness may function as an important candidate psychosocial determinant of youth mental health. Rather than operating solely through environmental exposure, nature connectedness appears to influence mental health through interconnected emotional, behavioral, and social processes embedded within adolescent development and social context. This conceptualization provides the theoretical basis for the three-pathway mechanistic model elaborated in the following section. The proposed framework is intended as an applied, mechanism-level elaboration for researchers and practitioners working at the intersection of environmental health, community and behavioral health, youth mental health prevention, and urban planning.

## 3 A three-pathway mechanistic model of nature connectedness

**Figure 1:**
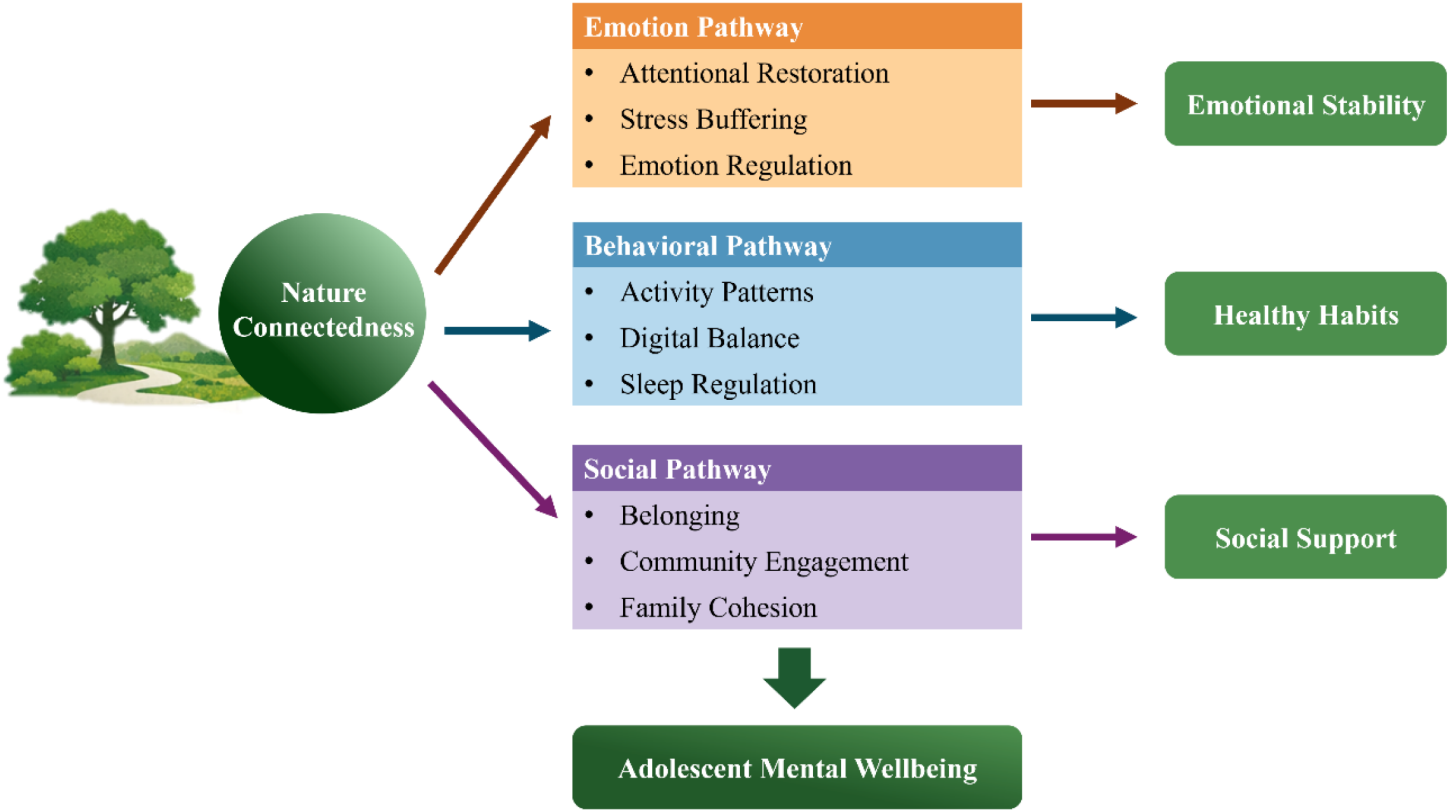
A three-pathway model of nature connectedness and adolescent mental health.

Building on the conceptualization of nature connectedness as a psychosocial determinant of adolescent mental health, we propose a three-pathway mechanistic model to explain how nature connectedness exerts its protective influence on youth well-being. While the emotional, behavioral, and social pathways may appear structurally parallel to the bio-psycho-social framework (35), the present model differs in its explanatory focus: rather than classifying determinants across biological, psychological, and social levels, it specifies relational and developmental mechanisms through which nature connectedness operates within and across these domains during adolescence. Biological processes such as stress physiology, autonomic regulation, and circadian functioning are conceptualized as dynamically embedded within these pathways rather than as separate domains. The model therefore offers a process-oriented and relationally grounded elaboration tailored to the dynamics of human–nature interaction in youth development (**Figure 1**).

### 3.1 Emotional pathway

Adolescence is marked by heightened emotional reactivity and ongoing maturation of regulatory systems, rendering young people particularly vulnerable to stress, anxiety, and mood disturbances (36, 37). Within this developmental context, we propose that nature connectedness may contribute to emotional stability through three interrelated regulatory processes: attentional restoration, stress buffering, and enhanced emotion regulation.

Attention Restoration Theory posits that natural environments replenish depleted cognitive resources by engaging involuntary attention, thereby reducing mental fatigue and improving executive functioning (38, 39). This restorative process is particularly salient for adolescents, whose academic pressures and intensive digital engagement place sustained strain on attentional systems. We hypothesize that a strong emotional and cognitive bond with nature may amplify these restorative effects by deepening adolescents’ engagement with natural environments, enhancing perceived psychological refuge, and strengthening the subjective experience of recovery (40, 41).

Stress Reduction Theory further suggests that natural environments can attenuate stress through both psychological and physiological pathways, including reductions in cortisol secretion, heart rate, and autonomic nervous system arousal (42, 43). For adolescents who are frequently exposed to chronic academic, social, and environmental stressors, nature connectedness may function as a readily accessible emotional resource that supports psychological containment and stress relief. Across experimental and observational studies, exposure to natural environments such as forests or tree-lined pathways has been associated with lower cortisol levels, slower heart rate, and more regulated autonomic nervous system activity compared with urban or non-natural settings (44–46). These findings support the plausibility of a stress-buffering pathway, although its precise causal structure remains to be empirically established.

Beyond stress buffering, existing evidence suggests that nature connectedness may also strengthen adolescents’ capacity for emotion regulation. Engagement with natural environments has been associated with greater emotional awareness, reduced rumination, and improved self-regulatory control (47). Experimental work indicates that even brief exposure to natural settings can reduce ruminative thinking relative to urban environments, while correlational studies link nature exposure to lower worry and more adaptive coping strategies (48). In addition, natural settings appear to promote present-moment awareness, which may further strengthen attentional regulation and deepen emotional insight (49). Collectively, these converging lines of evidence support a hypothesized emotional pathway through which nature connectedness may exert protective influences on adolescent mental health.

### 3.2 Behavioral pathway

The behavioral pathway delineates how nature connectedness may shape adolescents’ everyday behavioral patterns. Adolescence is a developmental period marked by increasing autonomy over lifestyle behaviors and heightened vulnerability to maladaptive habits, including physical inactivity, excessive screen use, and sleep disruption. These behavioral patterns are consistently associated with adverse mental health outcomes, including elevated symptoms of depression and anxiety and impaired psychosocial functioning (50–52). Within this developmental context, we propose that nature connectedness may function as a regulatory influence supporting healthier behavioral organization.

From a behavioral science perspective, established theories of health behavior emphasize the central role of intrinsic motivation and affective experience in sustaining long-term engagement in physical activity (53). Self-Determination Theory further proposes that behaviors are more likely to be initiated and maintained when they satisfy basic psychological needs for autonomy, competence, and relatedness (54). Consistent with these principles, existing evidence suggests that nature connectedness is associated with higher levels of physical activity among adolescents, particularly in outdoor and natural settings. Empirical studies indicate that youth with stronger emotional and relational bonds to nature engage more frequently in moderate-to-vigorous physical activity and often derive greater psychological benefits from such activity compared with indoor exercise (55, 56). Natural environments appear to provide intrinsically motivating contexts that enhance enjoyment, reduce perceived exertion, and foster sustained engagement in movement—factors that are especially relevant during adolescence, when physical activity levels typically decline (57, 58). Regular physical activity, in turn, confers well-established mental health benefits, including reductions in depressive and anxiety symptoms, improved mood, enhanced self-esteem, and more effective emotional regulation (59, 60).

Nature connectedness may also shape adolescents’ digital behavior patterns. A growing body of evidence suggests a reciprocal association between nature connectedness, outdoor engagement, and screen use, such that higher levels of screen time are associated with weaker emotional bonds with nature, whereas greater engagement in outdoor environments is linked to stronger nature connectedness (61). Excessive screen exposure is consistently associated with adverse psychological outcomes, while increased “green time” is linked to healthier emotional, cognitive, and social development (29, 62). These findings support a hypothesized process of behavioral rebalancing, in which adolescents who feel more connected to nature report lower levels of passive screen use and greater participation in outdoor, physically active, and socially interactive activities (55, 63). Evidence further suggests that screen time and outdoor time may operate in a compensatory relationship with meaningful implications for both physical and mental health (64).

Sleep disruption is highly prevalent during adolescence and constitutes a major risk factor for emotional dysregulation and the development of mental health disorders (65–67). According to the two-process model of sleep regulation, healthy sleep depends on the interaction between homeostatic sleep pressure and circadian timing, both of which are highly sensitive to behavioral and environmental influences during this developmental stage (68, 69). Within this framework, nature connectedness may contribute to healthier sleep through multiple interrelated mechanisms, including increased daytime physical activity, reduced evening screen exposure, and improved circadian alignment via regular exposure to natural light (70). Observational studies indicate that adolescents who engage more frequently with natural environments report better sleep quality, shorter sleep onset latency, and more regular sleep schedules (71, 72). Together, these converging lines of evidence support a plausible behavioral pathway linking nature connectedness with adolescent mental health, while underscoring the need for longitudinal and experimental studies to clarify causal structure.

### 3.3 Social pathway

The social pathway emphasizes the social dimension through which nature connectedness may influence adolescent mental health. According to Social Support Theory, perceived social support and the quality of interpersonal relationships constitute central protective factors for psychological wellbeing, buffering the negative effects of stress and reducing vulnerability to mental health problems (73). Supportive social environments provide emotional reassurance, instrumental assistance, and a sense of security, which are particularly salient during adolescence—a developmental period marked by rapid social and emotional transitions (30).

Belongingness represents a core psychosocial need, referring to the fundamental desire to feel accepted, valued, and meaningfully connected to others and to one’s social environment (74). This need becomes especially pronounced during adolescence as young people undergo profound changes in identity formation, peer orientation, and social role negotiation. Disruptions in belonging during this period have been shown to confer acute risk for severe mental health outcomes, including suicidal thoughts (75). Within this developmental context, natural settings may provide inclusive and non-competitive spaces that foster informal peer interaction, shared experiences, and cooperative activities, thereby creating developmentally supportive contexts for adolescent socialization (76, 77). In parallel, a substantial body of evidence demonstrates that stronger social connectedness is associated with enhanced feelings of belonging (78, 79). Although direct empirical evidence linking nature connectedness, social connectedness, and belonging within a single causal chain remains limited, the available findings support a plausible conceptual pathway in which nature connectedness may operate indirectly through social connectedness to strengthen adolescents’ sense of belonging and reduce vulnerability to mental health risk.

Beyond individual belonging, community engagement represents a broader social context through which nature connectedness may shape adolescent mental health. Community engagement refers to the process of bridging scientific knowledge and real-world practice through active community participation and collective action, with the goal of advancing health equity (80). Engagement with the community provides adolescents with a sense of purpose, social value, and collective belonging (81). In this regard, nature connectedness may function as a powerful catalyst for adolescent community engagement. Natural environments often serve as accessible and inclusive community spaces that facilitate informal social interaction, group-based activities, and intergenerational contact, including neighborhood recreation, school gardens, and community environmental programs (82, 83). Emerging evidence further suggests that youth involvement in nature-based community programs is associated with enhanced social competence, prosocial behavior, and psychological wellbeing (84, 85).

At the family level, family cohesion constitutes another critical social mechanism potentially linking nature connectedness with adolescent mental health. Higher levels of family cohesion have been consistently associated with lower emotional distress, fewer behavioral problems, and greater psychological resilience among adolescents (86). Shared engagement with natural environments may provide families with unique opportunities for meaningful interaction, joint activity, and emotional connection within low-distraction contexts. Empirical studies indicate that activities such as family walks, outdoor recreation, gardening, and other nature-based leisure pursuits are associated with more open communication, positive affect, and cooperative behavior, thereby strengthening emotional bonds and mutual understanding between parents and adolescents (87–89). Through repeated shared experiences in natural settings, families may cultivate more supportive relational climates characterized by trust, warmth, and effective communication, which in turn may buffer adolescents against psychological distress and support resilient mental health trajectories within the proposed three-pathway model.

## 4 Discussion

The present conceptual analysis advances a three-pathway model that reframes nature connectedness as a central psychosocial mechanism in adolescent mental health and situates this construct within a translational public health framework.

### 4.1 Nature connectedness as a modifiable public health target

Current adolescent mental health strategies remain heavily reliant on clinical services, individual psychotherapy, and digital interventions. While indispensable, these approaches are inherently constrained by high financial cost, workforce shortages, social stigma, and limited scalability, particularly in low-resource settings (90). In this context, nature connectedness represents a fundamentally different category of public health resource. It is not only comparatively low-cost and potentially scalable, but also modifiable at the population level through environmental design, educational systems, and community programming. Importantly, nature connectedness is not a fixed personality trait; it is a plastic psychosocial capacity that develops through repeated engagement, institutional practices, and social norms. This malleability positions nature connectedness as a strategic upstream target for primary prevention, rather than merely an adjunct to existing clinical services. By strengthening nature connectedness, public health systems may simultaneously influence emotional regulation, behavioral organization, and social integration—the three core domains underlying adolescent mental health vulnerability identified in this framework. Few currently available public health targets appear to offer comparable multi-domain leverage across developmental processes.

However, the scalability and equity potential of nature connectedness cannot be assumed without considering the structural conditions under which adolescents live and develop. Adolescents do not form relationships with nature within neutral or evenly distributed environments. Access to safe green spaces, environmental quality, outdoor educational opportunities, and culturally inclusive public environments is deeply structured by socioeconomic and spatial inequality (91–93). Moreover, these inequities extend beyond physical infrastructure alone. Time scarcity associated with economic precarity, limited school-based outdoor engagement, neighborhood safety concerns, experiences of discrimination in public space, and culturally mediated narratives about belonging in natural environments may all constrain adolescents’ capacity to form meaningful relationships with nature (94, 95). In this sense, access to nature is not solely a matter of proximity or availability, but also of social permission, psychological safety, and institutional support.

Recognizing these structural conditions reframes nature connectedness not as a universally available resource, but as a developmental capacity whose realization depends on environmental infrastructure, institutional practices, and policy choices. By conceptualizing nature connectedness as a form of relational access rather than mere environmental exposure, the three-pathway model explicitly links adolescent psychosocial development to broader structural determinants of health.

### 4.2 Translating the three-pathway model into interventions

Nature connectedness functions as a structurally embedded public health target whose relevance extends beyond conceptual articulation to concrete implementation within institutional and policy environments. The three-pathway framework provides a translational architecture for intervention design by explicitly mapping theoretical mechanisms onto applied contexts. Rather than remaining at the level of theoretical abstraction, the model offers a structured lens through which practitioners and policymakers may evaluate, design, and adapt real-world programs to ensure that emotional, behavioral, and social regulatory processes are meaningfully supported.

Within educational environments, for instance, the framework highlights how outdoor learning sessions, scheduled green breaks, or nature-based mindfulness practices may create opportunities for attentional restoration, stress buffering, and emotion regulation. At the level of behavioral organization, it encourages examination of how everyday institutional structures such as integrating outdoor movement into curricula, promoting active transport, or ensuring access to neighborhood green spaces may reshape adolescents’ patterns of physical activity, digital engagement, and sleep regulation. At the relational level, the model directs attention toward how community-based programs, family-oriented outdoor initiatives, youth environmental projects, and group-based activities in natural settings may cultivate belonging, peer cohesion, and family connectedness.

Globally, a range of established initiatives illustrates the practical feasibility of embedding structured nature engagement within institutional settings. Forest School programs implemented in the UK have progressed to feasibility cluster randomized trial evaluation, reflecting growing institutional and policy-level recognition of their potential to promote children’s emotional wellbeing (96). Beyond formal educational initiatives, mixed-methods studies of forest bathing among older adolescents have demonstrated significant increases in mental well-being, reduced stress, and enhanced positive affect following repeated participation (97). At the environmental design level, research on greening of school environments and biodiverse green spaces indicates improvements in cognitive functioning, attentional performance, and emotional outcomes among young people (98).

Although these initiatives were not explicitly developed according to a unified theoretical model of nature connectedness, their documented outcomes consistently span emotional restoration, healthier behavioral patterns, and strengthened relational engagement. This convergence across diverse settings provides indirect yet meaningful support for the plausibility of the mechanisms articulated in the present three-pathway framework and suggests that structured, sustained engagement with natural environments can generate multi-domain psychosocial benefits.

Crucially, this application-oriented perspective does not imply that programs should be artificially separated according to pathways. In practice, most real-world initiatives are organized around settings and lived experiences rather than abstract mechanisms. The three-pathway model therefore operates not as a prescriptive blueprint, but as an interpretive and design scaffold—clarifying which developmental processes are being activated, where regulatory gaps may exist, and how interventions may be refined to enhance coherence, sustainability, and long-term impact across educational, community, clinical, and policy domains.

### 4.3 Boundary conditions and implementation considerations

While the three-pathway framework highlights the integrative potential of nature connectedness for youth mental health, its developmental and policy impact is contingent upon several boundary conditions.

First, the effectiveness of nature-based strategies may depend on the quality and continuity of engagement rather than exposure alone. Brief or incidental contact with natural environments may not generate sustained psychosocial change unless opportunities for emotional reflection, behavioral integration, and relational embedding are actively supported. Program intensity, facilitation style, and institutional commitment may therefore moderate outcomes.

Second, developmental timing constitutes an important consideration. Adolescence encompasses substantial heterogeneity in emotional maturation, cognitive capacity, peer orientation, and identity formation. The regulatory and relational functions of nature connectedness may operate differently in early versus late adolescence, suggesting that age-sensitive implementation strategies are necessary.

Third, individual differences and contextual meaning systems may shape responsiveness. Prior experiences with nature, cultural interpretations of natural environments, gender norms, and perceived safety may influence whether adolescents experience nature as restorative, neutral, or distressing. For some youth, particularly those with histories of trauma or exclusion, certain natural contexts may not automatically confer psychological benefit.

Taken together, these considerations underscore that the impact of nature connectedness should be understood as context-dependent rather than universal. Effective implementation requires attentiveness to developmental stage, cultural context, and program design, ensuring that nature-based strategies are delivered in ways that are supportive, inclusive, and developmentally appropriate.

## 5 Conclusion

Nature connectedness represents a promising and adaptable conceptual framework for addressing the growing challenges of adolescent mental health. In this article, we provide a conceptual analysis of nature connectedness as a core psychosocial determinant of youth mental health, highlighting how these interrelated domains converge within a unified three-pathway model. By repositioning nature connectedness as a modifiable and structurally embeddable target, the framework moves beyond descriptive association toward actionable theory. It provides a coherent structure for understanding heterogeneity in adolescent responses to natural environments and offers a developmental lens for integrating nature-based strategies within educational, community, and mental health systems.

At the same time, the present framework remains conceptual and requires systematic empirical validation. Future studies should test the proposed pathways using longitudinal and experimental designs, quantify their relative contributions and interactive effects, examine boundary conditions across developmental stages and sociocultural contexts, and evaluate long-term outcomes. Parallel efforts are needed to develop rigorous implementation strategies, integrate nature-based approaches into public health systems, and build professional capacity to ensure that such interventions are delivered with scientific integrity, equity, and sustainability.
